# Membrane Deformation of Endothelial Surface Layer Interspersed with Syndecan-4: A Molecular Dynamics Study

**DOI:** 10.1007/s10439-019-02353-7

**Published:** 2019-09-13

**Authors:** Xi Zhuo Jiang, Liwei Guo, Kai H. Luo, Yiannis Ventikos

**Affiliations:** Department of Mechanical Engineering, University College London, Torrington Place, London, WC1E 7JE UK

**Keywords:** Mechanotransduction, Flow, Heparan sulfate, Lipid–protein interaction, Glycocalyx

## Abstract

The lipid membrane of endothelial cells plays a pivotal role in maintaining normal circulatory system functions. To investigate the response of the endothelial cell membrane to changes in vascular conditions, an atomistic model of the lipid membrane interspersed with Syndecan-4 core protein was established based on experimental observations and a series of molecular dynamics simulations were undertaken. The results show that flow results in continuous deformation of the lipid membrane, and the degree of membrane deformation is not in monotonic relationship with the environmental changes (either the changes in blood velocity or the alteration of the core protein configuration). An explanation for such non-monotonic relationship is provided, which agrees with previous experimental results. The elevation of the lipid membrane surface around the core protein of the endothelial glycocalyx was also observed, which can be mainly attributed to the Coulombic interactions between the biomolecules therein. The present study demonstrates that the blood flow can deform the lipid membrane directly *via* the interactions between water molecules and lipid membrane atoms thereby affecting mechanosensing; it also presents an additional force transmission pathway from the flow to the lipid membrane *via* the glycocalyx core protein, which complements previous mechanotransduction hypothesis.

**Electronic supplementary material:**

The online version of this article (10.1007/s10439-019-02353-7) contains supplementary material, which is available to authorized users.

## Introduction

The endothelial surface layer (ESL) shaping the intimal surface of blood vessels plays an important role in maintaining the vascular health and regulating biomolecular activities occurring on vessels.^27^ The ESL is composed of endothelial glycocalyx, soluble components generated by the endothelial cells and plasma components as well.^33^ Among the ESL components, the endothelial glycocalyx has gained an increasing attention from researchers due to its extensive involvement in vascular diseases.^29^ The glycocalyx is a complex layer of membrane-bound macromolecules^22^ including proteoglycans, glycoproteins and glycolipids.^27^ The glycocalyx features a highly negative charge imparted by its polyanionic constituents, such as glycosaminoglycan (GAG) side chains. The GAGs commonly associated with the vasculature are heparan sulfate (HS), chondroitin sulfate, and hyaluronic acid, with HS occupying 50–90% of the entire GAG pool.^23^ The transmembrane syndecans, the membrane-bound glypicans, and the basement matrix-associated perlecans constitute the major core proteins of HS proteoglycans found on endothelial cells.^24^ One of the hallmark functions of the endothelial glycocalyx surface is to transmit the mechanical signal from the blood flow stimuli into the cytoplasm. The mechanisms for the biomolecules sensing and transducing mechanical signals are referred as mechanotransduction.^30^ The correlation between the blood flow shear stress and the atherogenesis has been evidenced for half a century,^3^ which emphasizes the significance in studying the endothelial mechanotransduction.

Previous studies about endothelial mechanotransduction mainly focused on the contributions from the glycocalyx element.^26^,^28^,^34^ For example, Ebong *et al*. investigated the roles of HS chains in shear-induced endothelial nitric oxide synthase activation and cytoskeletal remodeling. Their results suggested that syndecan core proteins, due to their direct linkage to the cytoskeleton, transmit shear to the cytoskeleton in a decentralized mechanotransduction model.^8^ Besides the glycocalyx, the lipid membrane is also exposed to the blood flow, and can directly respond to shear stress by changing the lipid phase order and the fluidity.^36^ Organisms maintain the proper functioning of their membranes in response to various changes.^18^ Significant recent progress has enhanced our understanding of the molecular and cellular basis of lipid-associated biological functions from membrane trafficking to signal transduction.^18^ For instance, the lipid membrane does play a pivotal role in reorganizing of the actin cytoskeleton^32^ and acting as rafts to support the mobility of glycocalyx components.^38^ However, studies concentrating on the functionality of the lipid membrane in endothelial mechanotransduction are still lacking, leaving some fundamental questions unsolved. For example, how does the lipid membrane respond to the changes in vascular conditions (e.g. the changing blood velocity and the shedding of the glycocalyx)? Would the presence of the embedded glycocalyx element influence the behavior of the lipid membrane? What are the molecular/atomic mechanisms behind these phenomena? Solving these pending issues will have implications for understanding the pathologies for endothelium-related diseases and will potentially benefit the development of therapeutic strategies for pertinent diseases.

The present study aims to resolve these proposed questions using a molecular dynamics (MD) method. Specifically, an all-atom flow/glycocalyx/membrane system is constructed, and a series of scenarios are established to study the response of lipid membrane to the changes in vascular conditions in terms of deformation. The goal is to investigate the impact of blood flow velocity and the number of glycocalyx element on the lipid membrane deformation. Physiological implications of the lipid membrane deformation will also be discussed.

## Materials and Methods

### Model Description

The focus of this research is a small membrane patch of the endothelial cells. The membrane is a POPC (1-palmitoyl-2-oleoyl-sn-glycero-3-phosphocholine) lipid bilayer. Syndecan-4 (Syn-4) and HS chains are adopted in the construction of a simplified glycocalyx element, as Syn-4 is ubiquitously expressed and mediates numerous cellular processes like mechanotransduction^9^ and HS chains account for the majority of the GAG chains. An array of Syndecan-4 (Syn-4) core proteins are embedded into the lipid bilayer. The initial distance between every two adjacent individual Syn-4 core proteins is about 200 Å, which is in accordance with experimental observations.^2^ Every lumen end of the Syn-4 core protein is connected to six HS chains. The height of the EGL in the present model is 30–50 nm, originating from the upper membrane surface. The basal side of the membrane is exposed to the cell cytoplasm without considering the actin cortical web. The entire biomolecular system is solvated in a NaCl aqueous solution with a concentration of 0.1 M.

**Figure 1:**
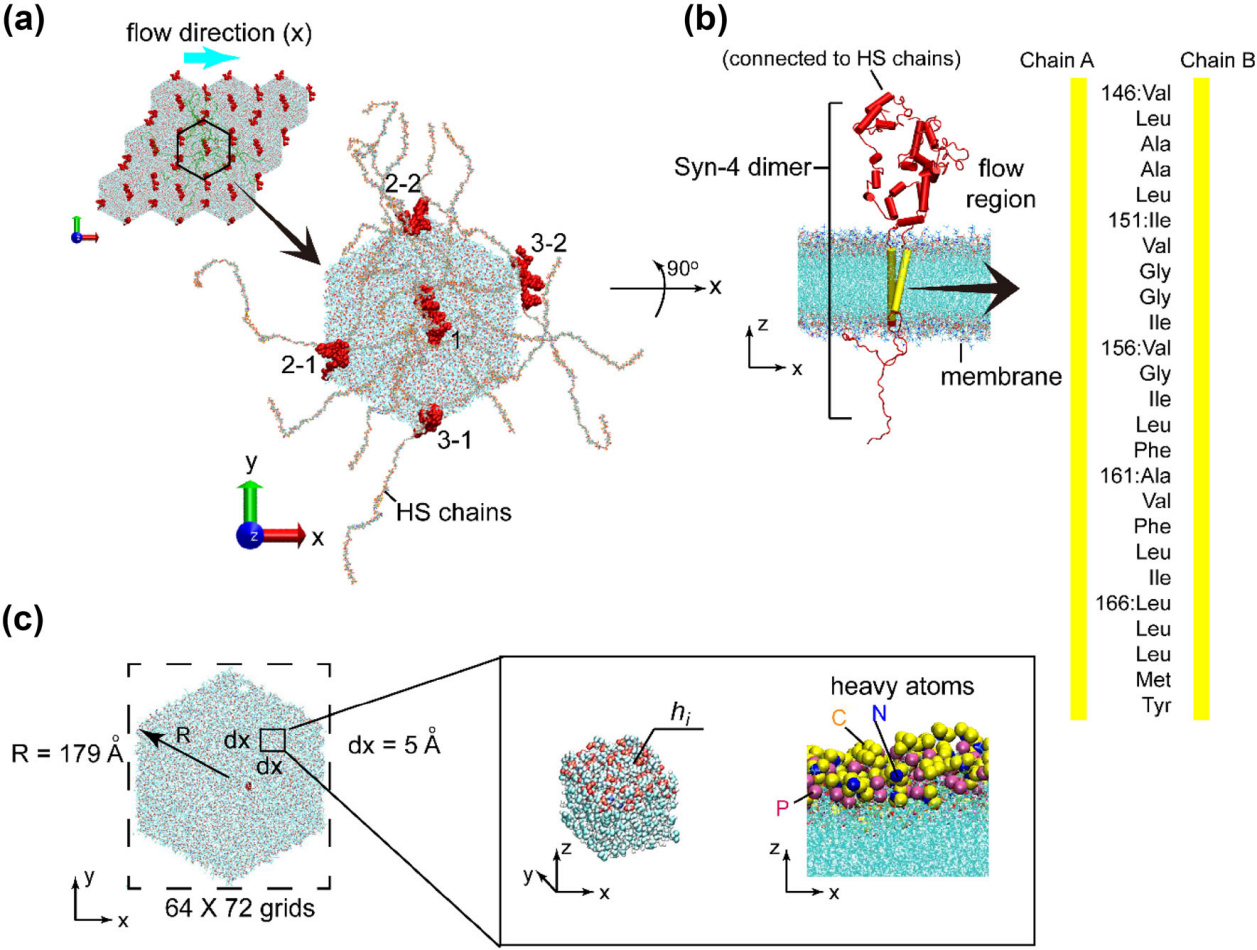
The atomistic model of the membrane interspersed with Syn-4 core proteins. (**a**) The hexagonal model unit with three Syn-4 core proteins. (**b**) Structural information of the Syn-4 dimer and amino acid sequence of the transmembrane chains. (**c**) Mesh used in the post-processing and grid information.

Figure 1a illustrates one unit of the proposed model. The membrane in the system was constructed in a hexagonal geometry, which is in accordance with previous studies.^34^,^38^ Three Syn-4 core proteins together with the attached HS chains were included in such a unit. Blood flow with a direction parallel to the membrane surface (*x*-direction) was simulated. It is noteworthy that there are indeed three core proteins in Fig. 1a, i.e. one central core protein labelled by 1, two marginal core proteins labelled by 2-1/2-2 and 3-1/3-2, respectively. The marginal two core proteins are visually separated into four parts due to the application of periodic boundary conditions. In the MD simulations, each of the three core proteins is a complete protein with a complete structure.

As shown in Fig. 1b, the Syn-4 core protein is a dimer, and the two chains of the dimer have identical transmembrane structures inserted into the POPC lipid bilayer. The sequence of amino-acid residues of the transmembrane parts is provided in Fig. 1b. As the focus of this research is on the interactions between the membrane and the core protein, structures tethered to the cytoplasmic end of the Syn-4 core protein is not constructed in the model. The HS length is assumed to be 100 sugar residues, with the end-to-end distance varying from 23 to 41 nm. More biochemical information about the Syn-4 and composition of HS chains can be found in a previous study.^5^

### Modelling Cases

The aim of this research is to explore the endothelial lipid membrane deformation under physiological/pathological flow conditions. Two aspects were considered in the design of biologically meaningful cases—the layout of Syn-4 core protein (i.e. the number of Syn-4 core proteins in a model unit) and the flow velocity, as these two factors are intimately related to vascular diseases.^4^,^31^

To mimic flow, external forces in the *x*-direction were imposed on each oxygen atom of water molecules in the ectodomain, and the tactic was successfully practiced in previous studies.^12^–^14^,^21^ By applying the same external force, a slip boundary between ectodomain flow and the upper membrane surface is assumed. Based on our previous findings, an external force of 0.003 fN would generate a laminar flow with a physiological bulk flow velocity.^14^ Thus, the case with an external force of 0.003 fN and three Syn-4 core proteins in a model unit was regarded as a physiological situation and used as a base case in the paper. The flow velocity is oscillating along the height within the ESL. For the small distance of ESL in nanometers, fluid shear stress is very sensitive to the fluctuations of the velocity. Thus, the value for the strength of the fluid shear stress is not provided here. To facilitate further discussion, the naming convention in this paper is clarified. Unless indicated, two numbers were used in the naming of a case: the first digital represents the number of the Syn-4 core proteins in a model unit, and the digitals after the underscore represents the strength of the imposed external forces. For example, 3_0.003 means there are three Syn-4 core proteins in the model unit and the external forces imposed on ectodomain water oxygens are 0.003 fN.

Five scenarios were constructed as summarized in Table 1. The first four scenarios were used to study the membrane deformations in response to the changes in Syn-4 quantity and the variations in the flow velocities. When the external force is set to 0, the system is supposed to mimic a stationary state with no flow. As mentioned previously, Case 3_0.003 represents a physiological situation and the longest physical time was simulated. Cases 3_0.01 and 1_0.003 represent two pathological situations with abnormal blood velocity and shedding of glycocalyx, respectively. Case 3_0 is a stationary setup for comparison only. The fifth case 3_0.003M is a virtual situation where the charges of atoms of the Syn-4 transmembrane amino acids (Fig. 1b) are artificially set to 0. By comparing results from Cases 3_0.003M and 3_0.003, the mechanism for the impact of core protein on lipid membrane can be explored.

**Table 1:** Summary of modelling cases in this study.

Cases	External force (fN)	No. of Syn-4	Physical time (ns)
3_0	0	3	8
3_0.003	0.003	3	30
3_0.01	0.01	3	15
1_0.003	0.003	1	15
3_0.003M^a^	0.003	3	15

^a^In this case, charges of atoms of the Syn-4 transmembrane amino acids are set to 0

### Modelling Details

The TIP3P water model^15^ was adopted to simulate water molecules. A CHARMM biomolecular force field^17^ was applied on the proteins and the lipid bilayer. For all the cases, an equilibrium simulation was first conducted at 1 atm and 310 K (NPT ensemble), using a Langevin thermostat and a Nosé-Hoover Langevin piston for 2 ns, followed by another simulation using a Langevin thermostat to maintain temperature at 310 K for 0.5 ns (NVT ensemble). The last frame of the NVT simulation was then used as the initial configuration of the follow-up “production” flow simulations. In the flow simulations, the Lowe-Andersen thermostat was selected to maintain the temperature at 310 K.

The velocity Verlet integration method^1^ was used to advance the positions and velocities of the atoms in time. A 2-fs timestep, and particle mesh Ewald^6^ electrostatics with a grid density of 1/Å^3^ are used. The SETTLE algorithm^19^ was used to enable the rigid bonds connected to all hydrogen atoms. The van der Waals interactions were calculated using a cutoff of 12 Å with a switching function starting at 10 Å.^5^

All MD simulations were performed using the software NAMD 2.9.^20^ The visualization of the molecular structures was performed by the VMD^10^ package. Non-visualized post-processing of the MD results was accomplished using Python (Python Software Foundation, Wilmington, De) scripts. All parallel simulations and non-visualized post-processing were conducted on ARCHER, UK’s national supercomputing service.

### Post-processing

The diameter of the circumcircle of hexagonal unit is 179 Å as illustrated in Fig. 1c. To quantify the deformation of the lipid membrane, the hexagonal membrane unit was enclosed by a rectangle with a dimension of 320 × 360 Å^2^ in the XOY plane. The rectangle was then meshed into grids with a dimension of 5 × 5 Å^2^. In each grid, the average *z*-position of heavy atoms (i.e. carbon, nitrogen, and phosphorus atoms) of the lipid heads was used to represent the height of the corresponding grid (i.e. *h*_*i*_). To facilitate the post-processing, the heights of the grids without membrane atoms were set to an unrealistic value. The time series of cases in Table 1 were sampled every 0.1 ns.

## Results

### Deformation of Lipid Membrane

**Figure 2:**
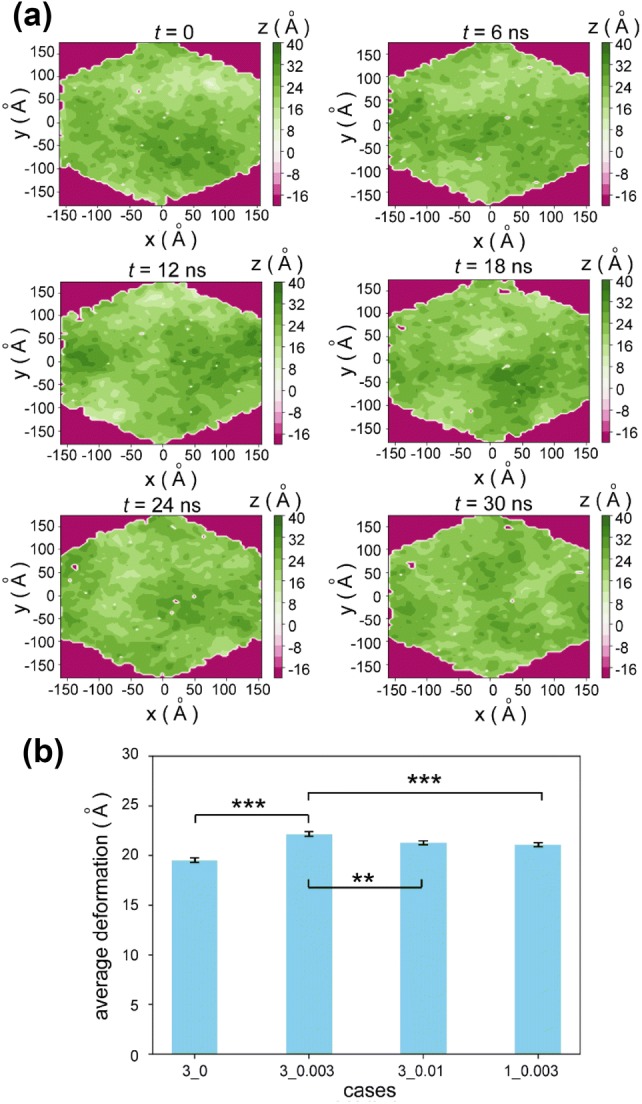
Height distributions and deformations of the lipid membrane upper surface in different scenarios. (**a**) Six consecutive snapshots of the lipid membrane upper surface. As flow passes, the height distribution changes (as shown in Supplementary Movie 1). (**b**) Deformations of the lipid membrane upper surface in the first four scenarios in Table 1. Generally, flow promotes the deformation of the lipid membrane, but the influence is non-monotonic. Case 3_0.003 is the base case for comparison. Statistics are given by mean ± SE, and significance is checked by ANOVA. ***p < 0.001 and **p < 0.01.

The deformation of the lipid membrane upper surface is mainly discussed in this paper as the upper surface is exposed to the blood flow. Case 3_0.003 is the base case for comparison, as it mimics a situation with blood flow velocity in a physiological range.^12^,^14^ Figure 2a illustrates six consecutive snapshots of the height distributions of the lipid membrane upper surface in Case 3_0.003. At the instant of *t* = 0, the surface of the lipid membrane is rough and coarse with varying heights at different locations. As flow passes through the surface, the hills and valleys of the lipid membrane surface migrate (The dynamic progress of the migration can be observed in the Supplementary Movie 1). The changes in the height distributions suggest that the flow results in the continuous deformation of the lipid membrane.

A set of valid and realistic heights, $\left\{ {h_{i} } \right\}$, were generated at each timestep. In the height set $\left\{ {h_{i} } \right\}$, the maximum five values were selected and averaged as the top value at the timestep. Analogously, the minimum five values were averaged as the bottom value. (The reported findings are not sensitive to the number of maximum/minimum values. Details can be found in Fig. S1.) The deformation of the lipid membrane upper surface at such timestep was defined as the difference between the top value and the bottom value. Using the difference between top and bottom values to quantify the deformation can distinguish two lipid membrane surfaces with the same spatial average heights, for the height distribution at each timestep can be approximated by a normal distribution as illustrated in the histograms in Supplementary Movie 1. To examine how the flow influences the lipid membrane deformation, the average deformations of the first four cases in Table 1 were calculated over time (Fig. 2b). Clarification should be made here: for Case 3_0.003, the average was conducted over the 30-ns simulation, and our data suggest that the effect of the external force on skewness accumulated with time can be neglected (as shown in Fig. S2); Case 3_0 is an equilibrated situation where quantities from the simulations are time-independent. Thus, time-average deformations can be compared among these four cases with different physical times. Generally, flow promotes the deformation of the lipid membrane. However, the influence of the blood flow velocity on the deformation is non-linear and non-monotonic. Specifically, deformation in Case 3_0.003, where a smaller flow velocity is generated, is greater than those in Case 3_0.01 and Case 1_0.003. An explanation for this phenomenon is provided in the “Discussion” section.

To gain additional insights into the lipid membrane surface deformation, the local surface of a grid is categorized into either hill or valley according to the deviation of its height, *h*_*i*_, from the global average height, $\bar{h}_{i}$, of the lipid membrane. ($\bar{h}_{i} = \varSigma h_{i} /n$, and *n* is the number of valid grid heights). If *h*_*i*_ is greater than $\bar{h}_{i}$ (i.e. $h_{i} - \bar{h}_{i}$ > 0), the grid is defined as hill; otherwise $\left( {h_{i} - \bar{h}_{i} \, < \,0} \right)$, it is valley. In accordance with the deviation from the global average height, the grids were further classified into three categories as follows:

Category I (large deformation): $\left| {h_{i} - \overline{h}_{i} } \right| \ge 8$ Å
Category II (medium deformation): $4 \le \left| {h_{i} - \overline{h}_{i} } \right| < 8$ Å
Category III (weak deformation): $0 < \left| {h_{i} - \overline{h}_{i} } \right| < 4$ Å

**Figure 3:**
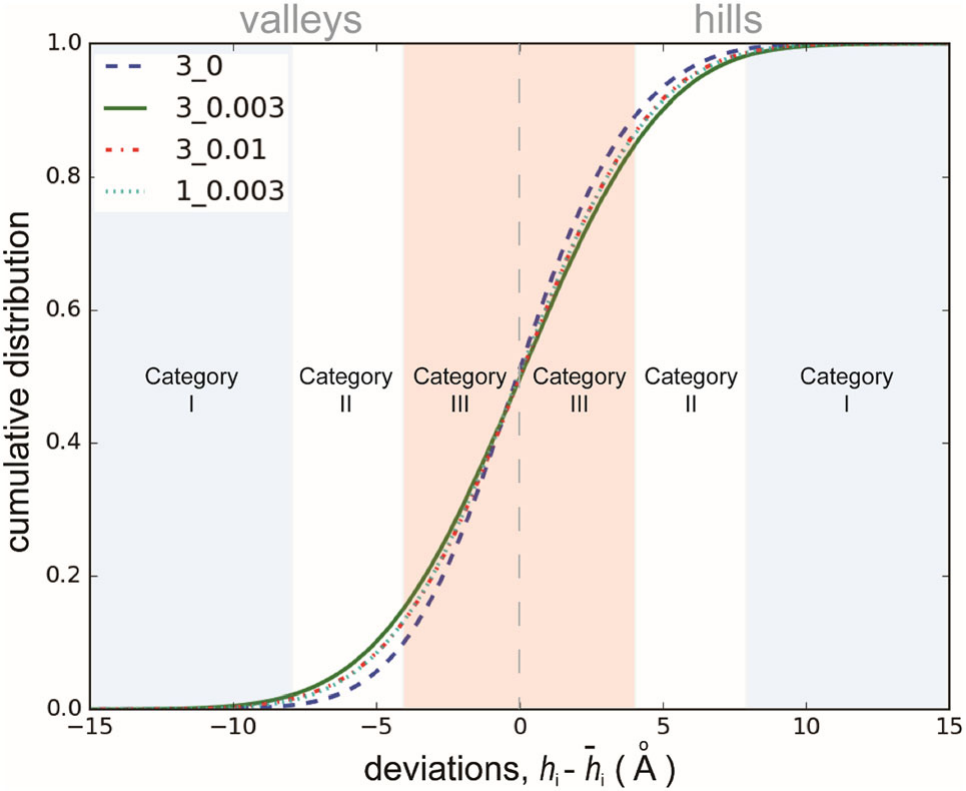
Cumulative distributions of hills and valleys of the deformed lipid membrane surface in four scenarios. Deformation of the lipid membrane can be divided into three categories: large (Category I), medium (Category II) and weak deformations (Category III).

The cumulative distributions for quantities of hills and valleys in terms of the three categories of the first four cases in Table 1 are summarized in Fig. 3. As expected, flow enhances the deformation of the lipid membrane surface, as the probabilities of large and medium deformations (i.e. Categories I and II) of the flow cases outnumber their stationary counterpart. In particular, Case 3_0.003 deforms the lipid membrane heavily. In the stationary case, the deformation is the weakest as the probabilities of weak deformations dominate.

### Strain Rate and Force

**Figure 4:**
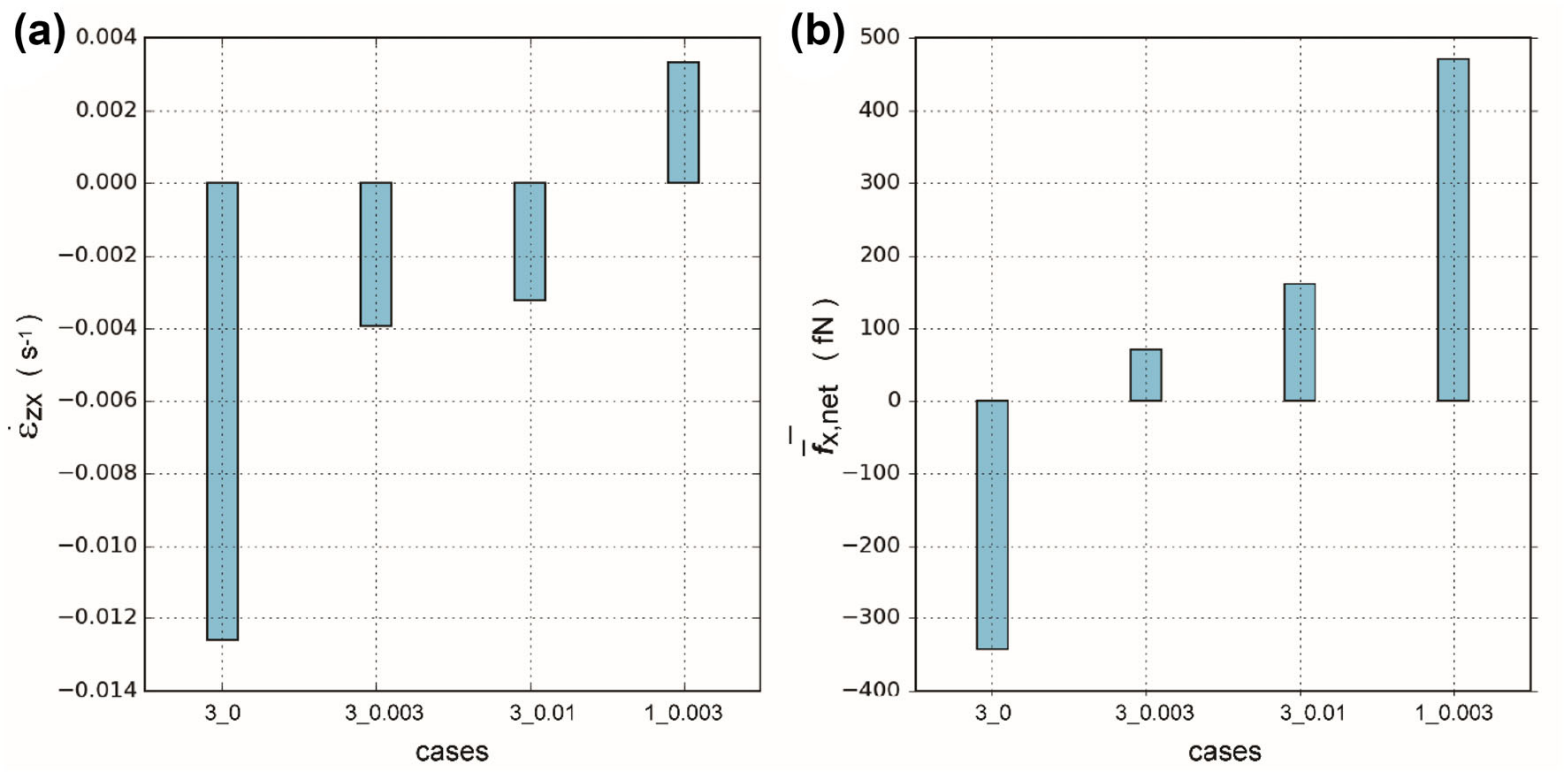
Means of strain rates and average net forces of the lipid membrane. (**a**) Means of the strain rates of the lipid membrane. (**b**) Means of the average net *x*-direction forces exerted on the lipid membrane atoms. (Distributions of the strain rates and the forces can be found in Figs. S3 and S4 in the Supplementary Information, respectively.)

To assess the deformation rate, strain rate of the lipid membrane was calculated. In the present research, one component from the strain rate tensor was selected and averaged to measure the deformation rate of the lipid membrane in the *XOY* plane in the direction of the blood flow. For a certain instant, the strain rate was computed in Eq. (1).

1 $$\dot{\varepsilon }_{zx} = {{\overline{v}_{{x,{\text{rel}}}} } \mathord{\left/ {\vphantom {{\overline{v}_{{x,{\text{rel}}}} } d}} \right. \kern-0pt} d}$$

where $\dot{\varepsilon }_{zx}$ is the average strain rate in the *x*-direction, and $\overline{v}_{{x,{\text{rel}}}}$ is the relative velocity between the two layers of the lipid membrane in the *x*-direction. In the calculation of the relative velocity, the velocities of the heavy atoms of the lipid membrane heads were used. *d* is the average thickness of the lipid membrane at the instant. In the present study, a constant value of *d* around 42 Å (will be mentioned in the Discussion) was used, a value which is supported by experimental observation.^7^ Furthermore, the average net force of the lipid membrane upper surface, $\overline{f}_{{x,{\text{net}}}}$, in the flow direction was calculated. (Details are described in Section S1 of the Supplementary Information). In the calculation of the average net force, the forces exerted on the heavy atoms of the lipid membrane heads were used. The means of both the strain rates and the average net forces for all the recorded timesteps in the four cases are illustrated in Fig. 4. (Distributions of the strain rates and the net forces are shown in Figs. S3 and S4, respectively, in the Supplementary Information.)

According to Eq. (1), the strain rate is in proportion to the *x*-direction velocity difference between the upper and lower lipid layers. The upper lipid layer is exposed to the flowing water molecules, whereas the lower lipid layer is in the static water condition. Thus, the velocity difference mainly depends on the velocities of upper lipid atoms. For the three cases with the three glycocalyx elements, the differences in relative velocities can be attributed to the varying external forces on water oxygens. Hence, an increase in $\dot{\varepsilon }_{zx}$ along the flow direction (i.e. $\dot{\varepsilon }_{zx} (3\_0.003)\, - \,\dot{\varepsilon }_{zx} (3\_0)\, > \,0$, and $\dot{\varepsilon }_{zx} (3\_0.01) - \dot{\varepsilon }_{zx} (3\_0.003) > 0$) can be observed in Fig. 4a as flow accelerates. In Cases 3_0.003 and 1_0.003 where the same external forces are imposed, a greater *x*-direction flow velocity can be expected in Case 1_0.003, due to the fewer obstacles therein. The larger momentum then transmits to the lipid membrane, resulting in a greater relative velocity and a higher strain rate in case 1_0.003 $\left( {\dot{\varepsilon }_{zx} (1\_0.003)\, - \,\dot{\varepsilon }_{zx} (3\_0.003)\, > \,0} \right)$. Water molecules exert force on the lipid membrane *via* interactions with lipid membrane atoms, thus, a similar trend of $\bar{f}_{{x,{\text{net}}}}$ in the four cases can be observed (Fig. 4b) as that in $\dot{\varepsilon }_{zx}$.

### Glycocalyx and Membrane Deformation

**Figure 5:**
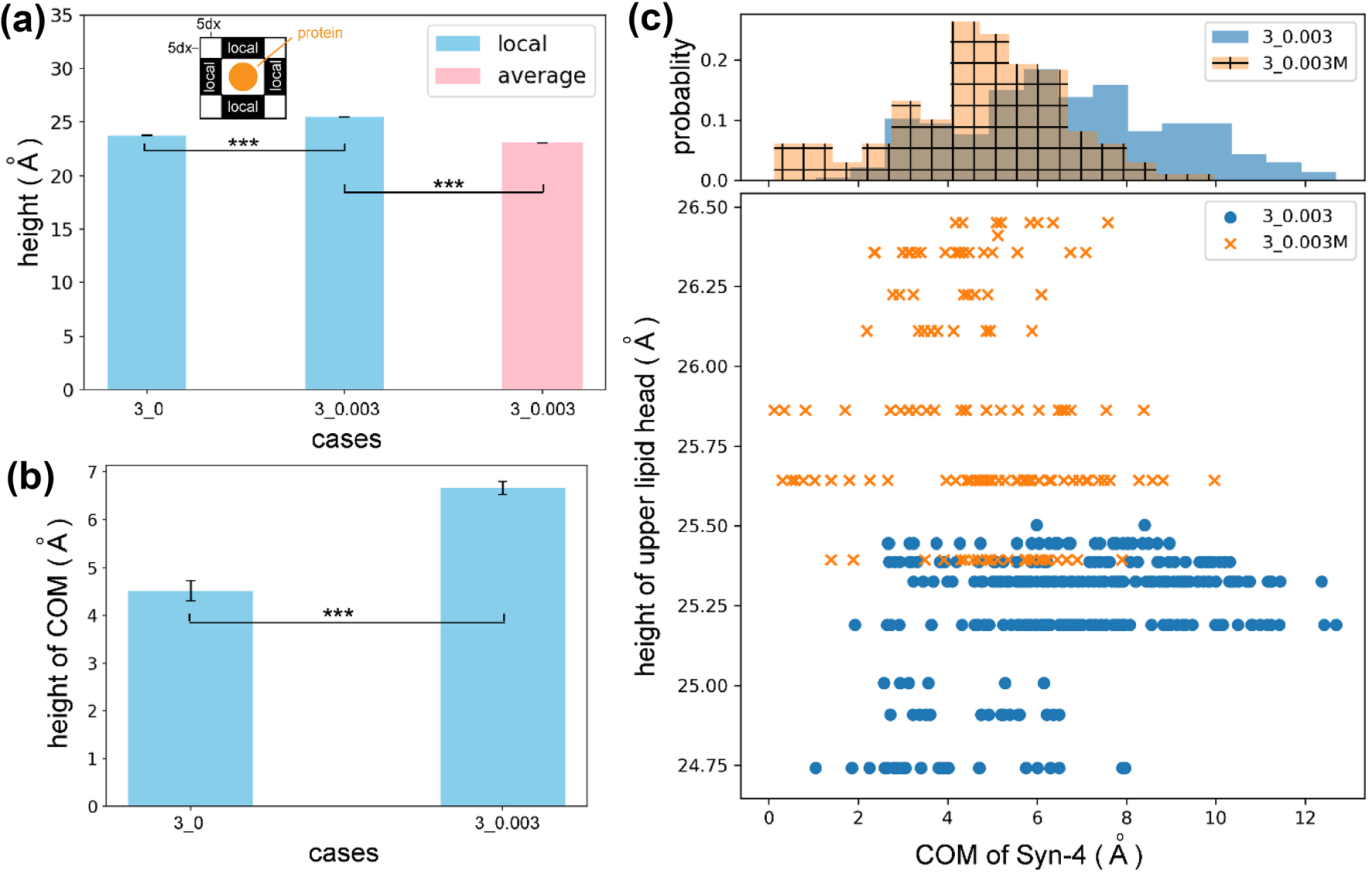
Syn-4 core protein and lipid membrane deformation. (**a**) Local height of the lipid upper layer head heavy atoms in close proximity to the central Syn-4 core protein and its global average over the upper surface. (**b**) Heights of COM of the Syn-4 core protein in cases with and without the flow. The core protein is elevated by the flow, and then lifts the surrounding lipid membrane. Statistics are given by mean ± SE, and significances are checked by ANOVA. ***p < 0.001. (**c**) Comparison of height of upper lipid head heavy atoms and COM of Syn-4 core protein between Cases 3_0.003 and 3_0.003M. The comparison implies that the Coulombic interactions play a dominant role in the deformation of lipid membrane caused by the Syn-4 core protein.

To determine how the glycocalyx element influences the membrane behavior, the local deformation of the lipid membrane in close proximity to the Syn-4 core protein was investigated. Take the central Syn-4 core protein as an instance. A rectangle with a dimension of 30 × 40 Å^2^ can be used to enclose the core protein. The heights of lipid head heavy atoms in the adjacent regions with five grids further to the rectangle (inset of Fig. 5a) were averaged, and the average height was used as a measure of the local membrane deformation around the core protein. For comparison, the average global height throughout the lipid membrane surface was calculated among all the valid grids. Figure 5a suggests that the Syn-4 core protein can lift the lipid membrane (comparison between local and average heights in Case 3_0.003), especially in the flow condition (comparison between Case 3_0 and Case 3_0.003).

To test our proposal about the lifting of the lipid membrane by the core protein, the centre of mass (COM) of the core protein was further scrutinized. As shown in Fig. 5b, the COM of the core protein is elevated by the flow, and the elevation can further cause the lifting of the surrounding lipid membrane.

When CHARMM force field is employed in the molecular dynamics modelling, the van der Waals and the Coulombic energies^17^ are considered to compute the nonbonded interactions between the lipid membrane and the Syn-4 core protein. To further explore individual contributions of the van der Waals and Coulombic forces from Syn-4 transmembrane part on lipid membrane deformation, Case 3_0.003M was conducted. As compared in Fig. 5c, when the Coulombic interactions are switched off, the height of the upper lipid head atoms increases whereas the COM of the Syn-4 transmembrane part declines. The contrast trends imply that the Coulombic interactions play a dominant role in the deformation of lipid membrane caused by the Syn-4 core protein.

## Discussion

### Deformations, Forces and Interactions

The deformation of the lipid membrane is a direct result of the forces exerted on the lipid atoms. Although the flow is only imposed in the *x* direction, the deformation of the lipid membrane occurs in all three directions as illustrated in Figs. 2 and 4 which depict the deformation of the upper lipid membrane in normal and shear directions. Surface deformation of the lower surface of the lipid membrane can also be observed, as shown in Fig. S5 in the Supplementary Information and Supplementary Movie 2. Consequently, the thicknesses of the lipid membrane at different locations vary with time as flow passes by. Indeed, in physiological flow conditions, the temporal changes in average lipid membrane thickness are not significant. We have calculated the average thickness over the lipid membrane for each timestep, and the probability density distribution of the thickness for all the timesteps suggests a thickness range of 42.0–42.3 Å, as shown in Fig. S6 in the Supplementary Information. The local thicknesses of individual grids over the lipid membrane are also recorded for every recorded timestep and the histogram suggests that the thickness about 42 Å is a representative value for the lipid membrane thickness as displayed in Supplementary Movie 3. Hence, in Eq. (1), a constant value for *d* is assigned. The influence of flow on lipid membrane deformation was also reported in a coarse-grained molecular dynamics study.^11^ The coarse-grained study explored the instabilities of the lipid membrane without the structures of core proteins under varying shear rates, thus, the rupture of the lipid membrane was observed when the shear rate increased. Our research aims to capture the performance of lipid membrane in the presence of endothelial glycocalyx. The interactions between the lipid membrane and the protein and their physiological implications are also within the scope of the study.

Figure 4 has attributed the upper surface deformation in the *XOY* plane (i.e. shear deformation) to the forces exerted in the pertinent plane. However, the impact from an external force is non-monotonic, especially on the *z*-direction deformations, as displayed in Fig. 2b. Here the lipid membrane can be regarded as a deformable surface subject to flow disturbances. At a certain flow rate, the deformable surface is expected to have a maximum deformation corresponding to its intrinsic frequency of oscillation. When the blood flow is continuously passing by at a physiological velocity (Case 3_0.003), the flowing water molecules may have exerted forces on the lipid membrane corresponding or close to such an intrinsic frequency, resulting in a greater lipid deformation than that in Case 3_0.01. The non-monotonic feature of the lipid membrane deformation and the proposed explanation agree with the viscoelastic model proposed by the experimental observations.^35^ Deformation, from the perspective of molecular dynamics, is the unsynchronized motion of two clusters of atoms.^14^ At the atomic scale, the *z*-direction velocity of the lipid membrane can be used as a measure of the *z*-direction deformation in each timestep. We have scrutinised the average *z*-direction velocities of the lipid membrane upper surface atoms in the three-glycocalyx-elements cases (in Fig. S7), and a greater average *z*-direction velocity is observed in Case 3_0.003, as expected. Compared with Case 3_0.003, the reduced two core proteins in Case 1_0.003 would lead to decreased interaction areas between the water molecules and the core proteins, and between the core proteins and the lipid membrane, thereby resulting in a weaker deformation. Details about the relationship between deformations and lipid-protein interaction are discussed in the following section.

### Deformations and Lipid–Protein Interaction

Figure 5 indicates an elevated local lipid surface around the Syn-4 core protein, which can be explained by a previous theory from the perspective of the hydrophobic properties of both the lipid membrane and the core protein biomolecules: the interior of the lipid membrane and the surface of the core protein transmembrane part are hydrophobic; to coexist with each other and the surrounding blood environment, the core protein has to alter its conformation or the lipid membrane has to distort to achieve dimensional match,^37^ especially when disturbance such as flow change occurs on the lipid membrane surface. In the present study, the COM of the core protein is elevated by the flow (Fig. 5b). To accommodate the core protein, the local lipid membrane must “climb”. Thus, an increase in the height of the local lipid membrane was observed (Fig. 5a). Indeed, our results further attribute the deformation to the Coulombic interactions between the lipid membrane and the core protein (Fig. 5c), which reveals the mechanism for lipid membrane deformation from the atomic perspective.

Both the blood flow and the Syn-4 core protein can cause the deformation of the lipid membrane. The contributions for both causes are roughly estimated as follows: Figure 2b suggests that the flow (Case 3_0.003) could cause ~ 1.4 Å extra deformation compared with the stationary case (Case 3_0) ((22.2 − 19.5)/2 = 1.4); Figure 5a indicates ~ 2.5 Å extra deformation is generated by the lifting of the core protein (26 − 23.5 = 2.5). In this regard, the deformation by the lifting of the Syn-4 core protein is stronger than that from the interactions with water molecules.

### Physiological Implications

The deformations of lipid membrane may have different physiological implications. The blood flow can deform the lipid membrane directly *via* the interactions between water molecules and lipid membrane atoms as reported in the present study and the aforementioned coarse-grained study.^11^ Such type of deformation can contribute to mechanosensing *via* affecting the gating of tethered channels.^25^

A previous theory proposed that a local torque generated by the glycocalyx core protein under flow shear stress causes the deformation of the actin cortical web and reorganization of the cytoskeleton thereby activating the signalling pathway.^32^ Such theory implies a mechanotransduction pathway that the load from flow shear stress is first transmitted to the glycocalyx core protein and then to the actin cortical web. However, whether the load is transmitted to the lipid membrane in the presence of the core protein is unclear based on their theory. Indeed, according to the present MD study, the load can be transmitted to the lipid membrane from the core protein, as the elevated COM of the core protein lifts the surrounding lipid membrane. In addition, when the two lipid monolayers are attached locally by transmembrane proteins, their relative slipping motion is restricted.^16^ The restriction can also benefit the collective deformation of the membrane, which contributes to the mechanotransduction.

### Limitations of the Simulations

As shown in Fig. 1a, an infinite endothelial glycocalyx surface is assumed due to the application of periodic boundary conditions in the *XOY* plane. Thus, the influences of intercellular junctions and cytoskeleton on membrane deformation are not considered in this research. An advanced model involving additional junctional and cytoskeletal information is desirable for further research.

To summarise, in this research, an all-atom molecular dynamics modelling was conducted to investigate the deformation of the lipid membrane of an endothelial glycocalyx surface. The model was built based on the experimental observation. A series of cases were set up to study how the lipid membrane deforms in response to changes in surrounding conditions. The spatial and temporal variations in the surface height suggest that flow results in the continuous deformation of the lipid membrane. By regarding the lipid membrane as a deformable surface subject to water flow disturbances, an explanation is provided for the different degrees of membrane deformation when either the flow velocity or the glycocalyx configuration is altered. The results also suggest that the strain rate of the lipid membrane upper surface depends upon the forces exerted on the lipid membrane surface atoms. In addition, lifting of the lipid membrane surface around Syn-4 core protein was observed as well, which can be attributed to the Coulombic interactions between the biomolecules therein. The present study reveals that the blood flow deforms the lipid membrane directly *via* the interactions between water molecules and lipid membrane atoms thereby affecting mechanosensing; it also presents an additional force transmission pathway from the flow to the lipid membrane *via* the glycocalyx core protein, which complements previous mechanotransduction hypothesis.

The current study sheds light on the mechanical functionality of lipid membrane in endothelial mechanotransduction, which enhances our knowledge about the atomic events occurring around the endothelial glycocalyx surface. Further studies are needed to clarify the impact of core protein types (e.g. glypicans and perlecans) on the lipid membrane deformation.

## Electronic supplementary material

Below is the link to the electronic supplementary material.

Supplementary material 1 (PDF 611 kb)

Supplementary material 2 (MP4 1956 kb)

Supplementary material 3 (MP4 1341 kb)

Supplementary material 4 (MP4 4676 kb)
