# Supplementary material for: Membrane Deformation of Endothelial Surface Layer Interspersed with Syndecan-4: A Molecular Dynamics Study
Source: Ann Biomed Eng. 2019 Sep 13;48(1):357–66. doi: 10.1007/s10439-019-02353-7 (PMC6928090; doi:10.1007/s10439-019-02353-7)
Supplement: Supplementary file 1 — Supplementary material 1 (PDF 611 kb) [file 10439_2019_2353_MOESM1_ESM.pdf]

Supplementary Information for

Membrane Deformation of Endothelial Surface Layer Interspersed with Syndecan-4: A Molecular Dynamics Study

Xi Zhuo Jiang†, Liwei Guo†, Kai H. Luo, Yiannis Ventikos**

Department of Mechanical Engineering, University College London, Torrington Place, London WC1E 7JE, UK

***Corresponding Authors**

Kai H. Luo and Yiannis Ventikos

Postal Address: Department of Mechanical Engineering, University College London, Torrington Place, London WC1E 7JE, UK

Supplementary Text

S1 Calculation of net force

The forces exerted on the heavy atoms of the lipid heads of both layers were provided by the results from NAMD (.forcedcd file). The net force is the vector summation of the x-direction forces of all the heavy atoms of the lipid heads. The average net force, $\overline{f}_{x,\mathrm{net}}$, used in Fig. 4 (main text) is the net force divided by the number of heavy atoms of the lipid heads. It is noteworthy that the average net force comprises two parts: one from atoms/molecules outside the lipid membrane which is influenced by the impulse from the external forces imposed on the water molecules; the other from the atoms within the lipid membrane which are mainly the intra-molecular interactions to maintain the stable structure of the lipid membrane and can be regarded as constant.

Supplementary Figures

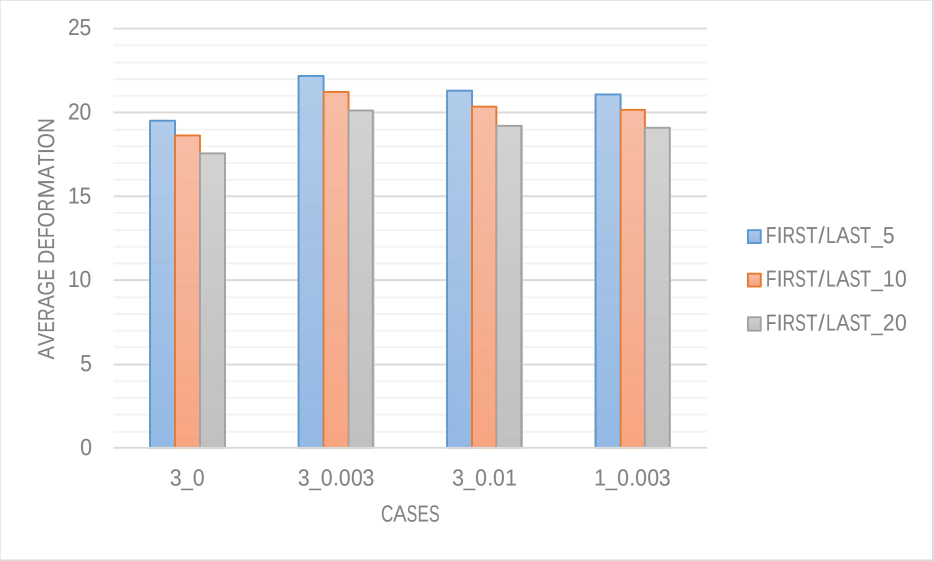

Fig. S1 Average deformations for the four cases in Table with varying number of maximum/minimum values. Comparisons suggest that the reported findings are not sensitive to the number of maximum/minimum values. The average deformation decreases as the number of maximum/minimum values increases. To highlight the deformations, we selected 5 maximum/minimum values to quantify the deformations in the main text (Fig. 2b).

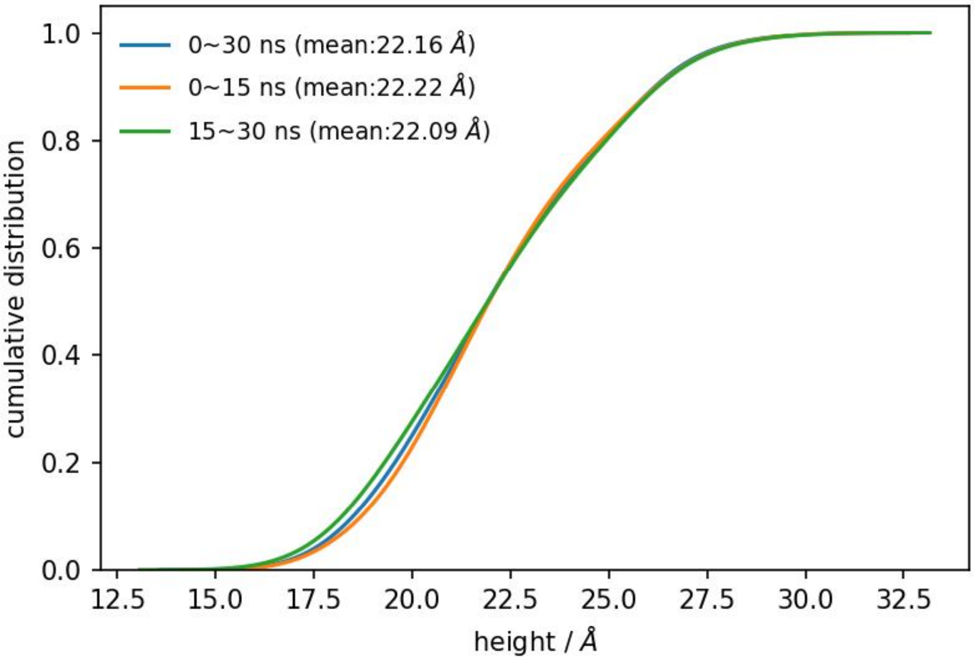

Fig. S2 Cumulative distributions and means for deformations at different time intervals in Case 3_0.003. (Means are shown in the legend.) The close cumulative distributions and the mean values suggest that the skewness by the numerical transient effects from the application of an external force can be neglected.

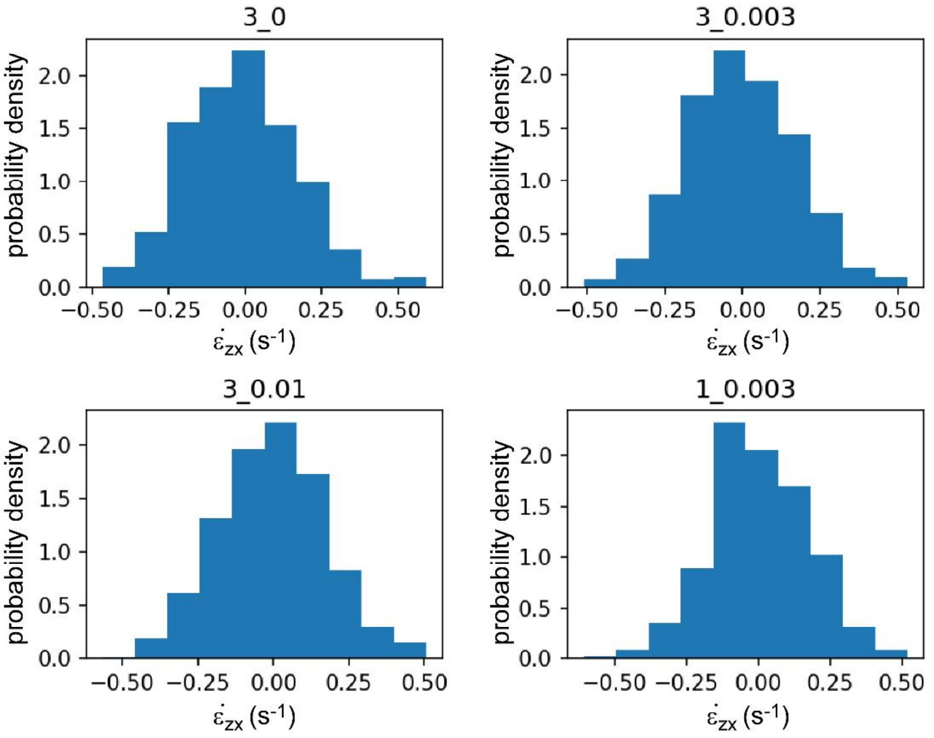

Fig. S3 Normalized distributions of *x*-direction strain rates, $\dot{\varepsilon}_{zx}$, for all the recorded timesteps in the four cases. For each recorded timestep, a strain rate is generated. The probability density distributions are calculated among all the recorded timesteps.

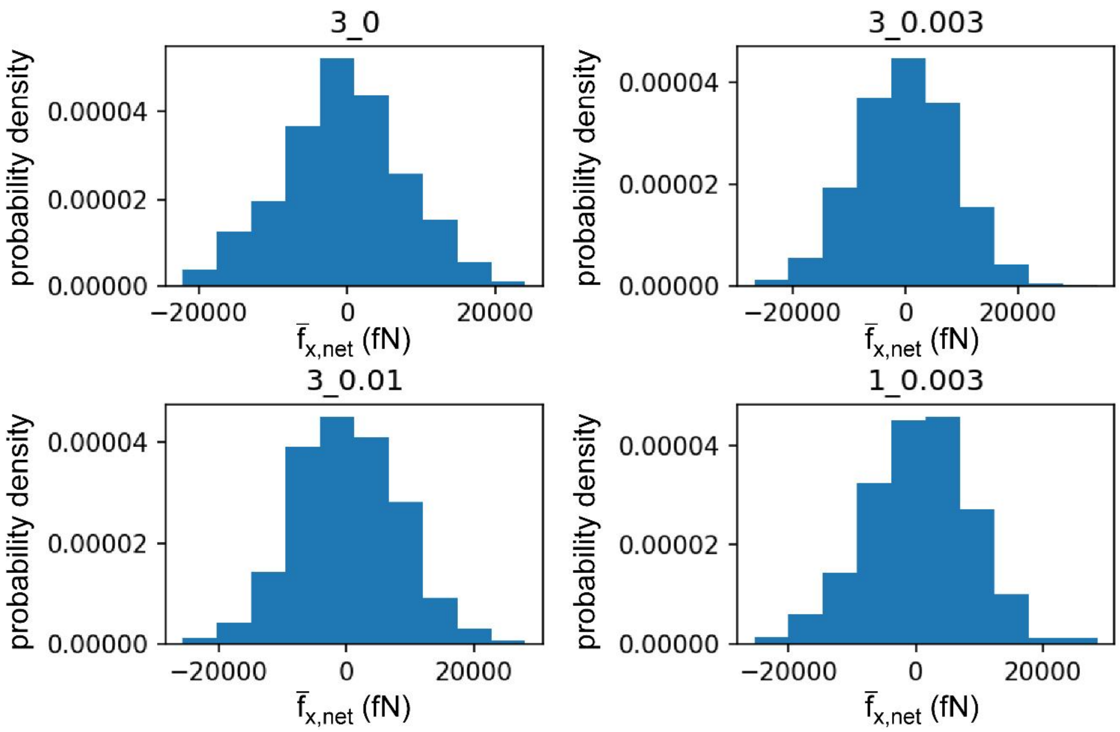

Fig. S4 Normalized distributions of *x*-direction net force per heavy atom for all the recorded timesteps in the four cases. For each recorded timestep, the *x*-direction forces are averaged among the heavy atoms of the upper lipid membrane. The probability density distributions are calculated among all the recorded timesteps.

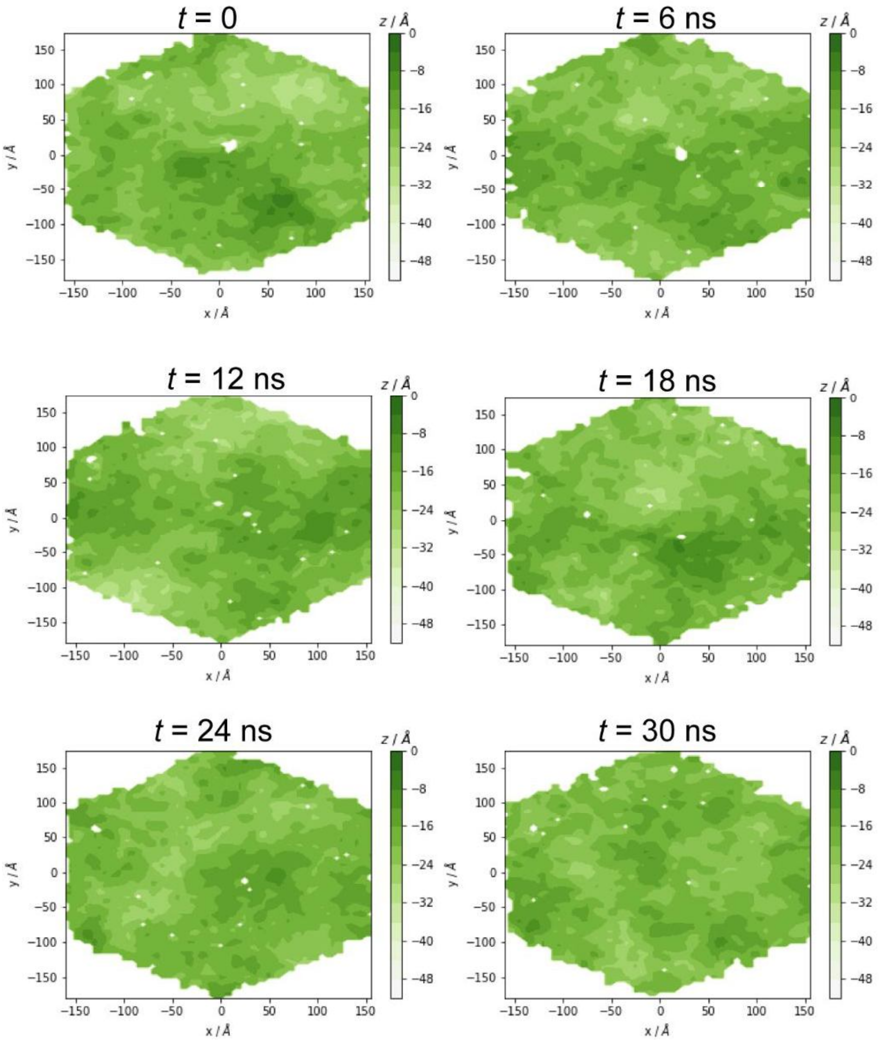

Fig. S5 Deformation of the lower lipid membrane surface.

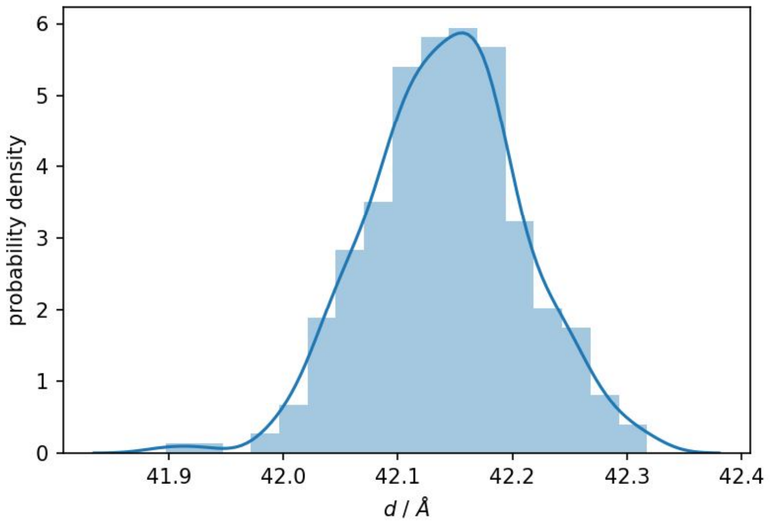

Fig. S6 Probability density distribution of the average thickness of lipid membrane for all recorded timesteps. The thickness of lipid membrane is in the range of 42.0~42.3 Å.

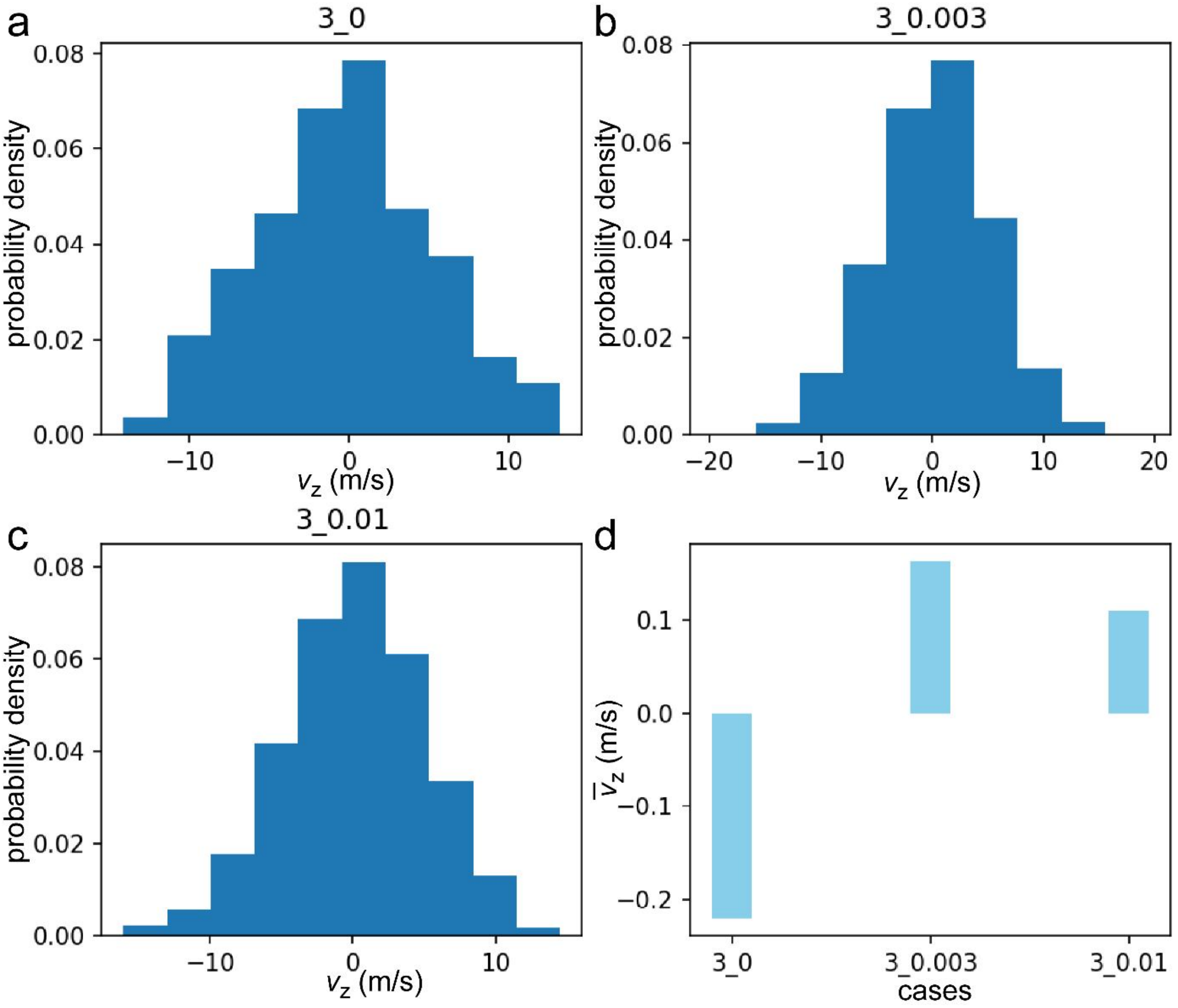

Fig. S7 Average z-direction velocities on the upper lipid surface atoms. a.-c. Probability density distributions for *z*-direction velocities for the three-glycocalyx-element cases. For each recorded timestep, the *z*-direction velocities are averaged among the heavy atoms of the upper lipid membrane. The probability density distributions are calculated among all the recorded timesteps. d. Mean values of the average *z*-direction velocities in a-c. Statistical differences are not significant.
